# Treatment of Aggressive NK-Cell Leukemia: A Case Report and Review of the Literature

**DOI:** 10.1155/2011/818469

**Published:** 2011-11-21

**Authors:** Anders Kindberg Boysen, Paw Jensen, Preben Johansen, Karen Dybkær, Mette Nyegaard

**Affiliations:** ^1^Department of Hematology, Aalborg Hospital, Aarhus University Hospital, Hobrovej 18-22, P.O. Box 365, 9100 Aalborg, Denmark; ^2^Department of Pathology, Aalborg Hospital, Aarhus University Hospital, 9100 Aalborg, Denmark

## Abstract

Aggressive NK-cell leukemia is a rare malignancy with neoplastic proliferation of natural killer cells. It often presents with constitutional symptoms, a rapid declining clinical course, and a poor prognosis with a median survival of a few months. The disease is usually resistant to cytotoxic agents, and no treatment has emerged as the standard of care for these patients. We report a case of an 18-year-old male who obtains complete remission following two lines of combination chemotherapy. We describe in details our regimens for induction chemotherapy and perform a review of existing literature concerning treatment of aggressive NK-cell leukemia.

## 1. Introduction

Aggressive NK-cell leukemia (ANKL) is a highly aggressive disease, which has only been recognized as a clinical entity in the last 20 years [1]. It is a rare malignancy accounting for less than 1% of all non-Hodgkin lymphoma in Europe and North America, being more prevalent in Asia and Latin America [2]. ANKL often affects young patients and is characterized by relative resistance to standard chemotherapy, association with Epstein-Barr virus infection [3], and a dismissal prognosis with a median survival less than 2 months [4]. Due to the rarity and often rapid fatal course of this disease, no randomized prospective clinical trials have been performed, and the therapeutic principles are based on case reports and small retrospective cohorts of patients [5]. We report a case of a young man with ANKL, who achieved complete remission following chemotherapy. Allogeneic transplantation was performed, but the patient experienced an intractable relapse eight months later. 

## 2. Case Presentation

An 18-year-old male with no prior medical history was admitted to the Department of Medical Gastroenterology in April 2009. The symptoms were nausea, vomiting, diarrhea, fatigue, and a weight loss of 11 kg in the last 3 weeks. Physical examination revealed an enlarged liver and spleen, and biochemistry showed marked increased level of the enzyme Lactatdehydrogenase (LDH) to 4650 U/L (ref. 105–205 U/L) and low platelet count of 64∗10^9^/L (ref. 145–350∗10^9^/L). The white blood cell count was normal, and the hemoglobin level was 7.9 mmol/L being just below the reference level (ref. 8.1–10.3 mmol/L). A hematological neoplasm was suspected, and the patient was transferred to the Department of Hematology for further investigation. Computed tomography (CT) scan revealed enlarged lymph nodes of the neck and chest cavity measuring up to 2.2 cm, a retrosternal tumor of 6 cm suspected of being a thymoma, and an enlarged spleen and liver. Blood and bone marrow examination showed peripheral blood with 15% blastic cells and bone marrow smears with 80% blastic cells. Flow cytometry showed the following phenotype: Sm: CD 3^−^, Cy CD3^+^, CD2^+^, CD7^+^, CD56^+^, and CD34^+^ (partially); there was negative reaction for TDT, MPO, CD117, CD20, CD79A, and CD57. A diagnosis of ANKL was made. Section from the bone marrow showed a densely packed marrow.

The initial treatment strategy was combination chemotherapy and in case of response a consolidating allogeneic bone marrow transplantation (BMT). The chosen chemotherapy regimen was related to the induction chemotherapy of acute lymphoblastic leukemia and consisted of cyclophosphamide (1200 mg/m^2^ D1), vincristine (1.4 mg/m^2^ D1, 8, 15, 22), daunorubicin (45 mg/m^2^ D1–3), prednisolone (60 mg/m^2^ daily), and L-asparaginase (5.000 IE/m^2^ D15–24) in combination with intrathecal administration of methotrexate (10 mg/m^2^ D1). Prophylactic against hyperuricaemia allopurinol 300 mg was added.

**Figure 1:**
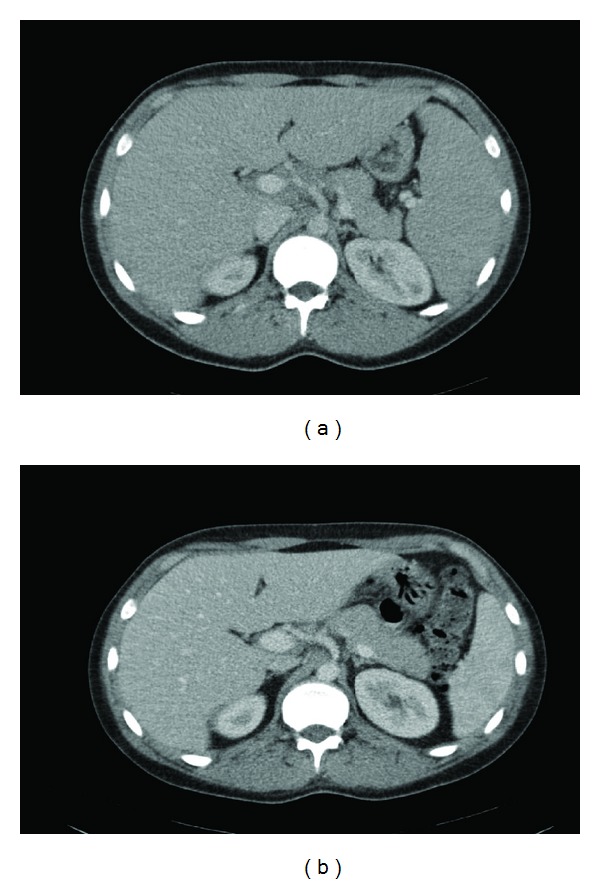
CT scan of the abdomen before (a) and after (b) first line of chemotherapy.

In May 2009, a second bone marrow examination was performed, and the infiltration of the leukemic cells had diminished from initially 80% to 1-2%, so the patient had a partial response, with the residual leukemic cells having the same immunophenotype as initially. The condition also improved clinically with stabilization of the weight loss and a satisfactory biochemistry with a normalized platelet count, white blood cell count, and a slightly decreased hemoglobin level. CT scan showed normalization of the hepatosplenomegaly (Figure 1). A new bone marrow examination was performed in June 2009 followed by a second course of chemotherapy. The infiltration of leukemic cells had increased to 5–10%. Second-line chemotherapy regimen included high-dose methotrexate (3 g/m^2^ D1), ifosfamide (1500 mg/m^2^ D1–3), etoposide (100 mg/m^2^ D1–3), and L-asparaginase (6.000 IE/m^2^ D8–20), with mesna as urothelial protection. In July 2009, examination of the bone marrow showed complete remission, and the patient was referred to Rigshospitalet, Copenhagen, for allogeneic BMT, with his mother being utilizable as a donor. The BMT took place in August 2009, and the myeloablative conditioning was total body irradiation and high-dose etoposide (60 mg/kg). Besides the expected side effects such as severe mucositis and nausea, the course was complicated by the development of a graft versus host (GvH) reaction in the skin treated with immunosuppressives, onset of thrombotic thromobytopenic purpura, on a microangiopatcic basis, and CMV infection. 

In March 2010, the patient developed pancytopenia and increasing levels of LDH. A bone marrow examination was performed, showing a relapse of ANKL with an infiltration of 70–80%. Apart from recurrent fever and infectious complications to the central line catheter, the patient was relatively well and in acceptable performance status. It was decided to attempt a chemotherapy regimen consisting of high-dose methotrexate (3 g/m^2^) and L-Asparaginase (6.000 IE/m^2^) which had shown effect in the setting prior to the BMT. After one month of treatment, a new bone marrow examination was done in April 2010, indicating no major effect of the treatment, with the bone marrow infiltrated with 60% NK leukemic cells, and the treatment stopped. At this point, no further chemotherapy was administered, and the strategy from here on was best supportive care. The patient died from sepsis and multiorgan failure in May 2010, 13 months after the debut of the disease.

## 3. Discussion

ANKL is a potentially chemosensitive disease, and complete remission might be reached though no chemotherapy-regimen has yet emerged as the standard of care for these patients [6]. In the literature, some guidance regarding the chemotherapeutic agents exists. L-Asparaginase has shown promising results against NK cell neoplasms [7, 8], and one study has demonstrated L-Asparaginase-induced apoptosis of NK cell tumours *in vitro* [9]. The activity of etoposide against NK cell malignancies has been reported both *in vivo* and *in vitro* [10, 11]. In the case described above, our choice of treatment was inspired by the phase I study by Yamaguchi and colleagues [12] of 6 patients with NK cell lymphoma treated with the SMILE regimen (steroids, methotrexate, ifosfamide, L-Asparaginase, and etoposide) showing a response rate of 67% and 50% complete response. The role of stem cell transplantation in the treatment of NK cell leukemia has been studied by the NK-cell Tumor Study Group [13] who performed a survey of 40 patients with NK-cell lymphoproliferative diseases treated with either allogeneic (*n* = 15) or autologous (*n* = 25) stem cell transplantation. The results indicate a beneficial effect of stem cell transplantation, but the criteria for transplantation were not uniform, and the patient population was heterogeneous with only 3 patients having ANKL as underlying disease.

A comprehensive review of all published cases of NK-cell leukemia in English literature has been performed by Ruskova and colleagues [14] analyzing 73 cases from 1966 to 2003. They reported an epidemiological profile with a median age of 37 years at diagnosis and a slight male predominance, alongside a clinical picture with acute onset of symptoms and splenomegaly which is in concordance with the patient presented in our paper. The median survival was 61 days, compared to 13 months in the presented case.

Our case demonstrates that it is possible to treat this rare and highly aggressive disease and to obtain complete remission following intense chemotherapy. Although the consolidation BMT failed to achieve curability, the overall treatment strategy was effective in prolonging the time to progression, reducing the severe systemic symptoms and improving the survival as compared to the expected median survival of 2 months, referred to in the literature [14].
